# Rhopressa-induced corneal edema: a case report

**DOI:** 10.1186/s13256-021-02665-0

**Published:** 2021-04-02

**Authors:** Matthew J. Chu, Michael Song, Trisa Palmares, Alice Song, Julia Song

**Affiliations:** 1 Southern California Eye Physicians & Surgeons, 800 S. Fairmount Ave., #207, Pasadena, CA 91105 USA; 2 Center for Oculofacial and Orbital Surgery, 3771 Katella Ave., #209, Los Alamitos, CA 90720 USA

**Keywords:** Glaucoma, Netarsudil (Rhopressa), Corneal edema, Rho kinase inhibitors

## Abstract

**Background:**

Rhopressa (netarsudil) has recently been added to the arsenal of treatment for open-angle glaucoma. It is an effective norepinephrine transporter and Rho-associated protein kinase (ROCK) inhibitor used to decrease intraocular pressure (IOP), with the most common side effect being conjunctival hyperemia.

**Case presentation:**

We report a unique case of Rhopressa-induced corneal edema in a 79-year-old African-American woman, which resolved after discontinuation. She had a history of smoking one cigarette per day and did not consume alcohol. She had no history of corneal edema or uveitis.

**Conclusions:**

Previous case reports have documented patients with Rhopressa-induced corneal edema; however, they have all had a preexisting history of corneal edema or uveitis. We believe that this is a unique case of Rhopressa-induced corneal edema in a relatively healthy eye. While Rhopressa is effective in managing glaucoma, there may be effects of treatment that are still unknown. We will discuss clinical findings of our case, along with a review of previous literature on Rhopressa and novel ROCK inhibitors. We hope that we can add to the existing body of literature and invite further investigation of Rhopressa and ROCK inhibitors and their effects on the cornea.

## Background

Glaucoma is one of the most common eye disorders leading to blindness worldwide. Treatments include topical and oral medications, laser therapy, and surgery. A new class of topical medications has recently become available that directly targets the trabecular meshwork. Rhopressa (netarsudil) was developed by Aerie Pharmaceuticals and has been approved by the United States Food and Drug Administration (FDA) for lowering intraocular pressure (IOP) in patients with glaucoma [1]. It is a Rho-associated protein kinase (ROCK) and norepinephrine transporter inhibitor, resulting in increased outflow from the trabecular meshwork, reduced aqueous humor production, and decreased episcleral venous pressure [1]. The inhibition of norepinephrine causes constriction of the vascular structures of the eye, leading to decreased blood flow to the ciliary processes and inhibition of aqueous humor production [2].

Typical adverse events (AE) are noted as mild conjunctival hyperemia and minimal systemic AEs. Other common side effects include corneal verticillata (deposits with swirling appearance), eye pain, and subconjunctival hemorrhage. Infrequent side effects include decreased visual acuity (VA), blurred vision, excessively watery eyes, eyelid erythema, and staining of the cornea [1]. Rare side effects include corneal inflammation due to bacterial infection.

Rhopressa is effective in lowering IOP, and ROCK inhibitors in general have been shown to have a wide range of benefits including direct effects on the trabecular meshwork [3]. Other benefits include neuroprotection of the optic nerve head, improved ophthalmic perfusion, decreased inflammation, prevention of scarring following glaucoma filtration procedures, and improved corneal healing [4]. There have also been positive effects of ROCK inhibitors specifically on the corneal epithelial membrane, as was seen in studies of corneal endothelial wound healing in both rabbit and primate models [5, 6]. The same authors conducted a trial of ROCK inhibitors for patients with Fuchs’ endothelial corneal dystrophy, which was successful in preserving corneal clarity and VA [5, 6].

The reviewed literature has shown positive results from the use of ROCK inhibitors on other parts of the eye, but there exist few cases of corneal edema induced by Rhopressa. A recent review reported that patients treated with Rhopressa had episodes of reticular bullous epithelial corneal edema which improved upon discontinuation of the medication [7]. These patients had a history of either corneal edema or uveitis in the affected eye. In another case study, one patient presented with failed Descemet’s stripping automated endothelial keratoplasty (DSAEK) and stromal edema [8]. After treatment with Rhopressa for 5 days, the patient developed severe reticular epithelial edema. After discontinuation of Rhopressa, the patient underwent repeated keratectomy and DSAEK, with VA improvement from hand motions to 20/200. In this same report, the authors describe a patient with a partially detached cornea. The patient was started on Rhopressa, reticular epithelial edema occurred 11 days later, and on day 15, the patient’s epithelium and stroma cleared [8].

There are different effects of ROCK inhibitors on the cornea, inviting the question of Rhopressa's effect  on the cornea. We present the case of a 79-year-old woman with advanced chronic angle-closure glaucoma who started Rhopressa due to worsening visual field defects OS. She complained of ocular pain and decreased VA OS within the first week of Rhopressa use, due to corneal edema. Therefore, Rhopressa was discontinued. After discontinuation, there was improvement in VA and decreased ocular pain. Previous cases described patients with a history of corneal edema or uveitis who developed corneal edema following Rhopressa use. Our patient had no such known history of corneal edema or uveitis. This is the first reported case of Rhopressa-induced corneal edema in an otherwise healthy eye.

## Case presentation

Our patient was a 79-year-old retired African American woman with advanced angle-closure glaucoma in both eyes (OU). Her past medical history was significant for diabetes, hypertension, and gastroesophageal reflux disease. Past ocular history was significant for laser iridotomies OU, bilateral upper lid ptosis repair, cataract extraction with intraocular lenses OU, mini-shunt OS, selective laser trabeculoplasty (SLT) OS, bilateral lower lid punctal cautery, laser capsulotomies OU, and Baerveldt glaucoma tube implants OU. Her medications for diabetes included insulin glargine 100 U/mL injection every night at bedtime, humalog injection for blood glucose over 150 mg/dL, metformin 500 mg tablet twice daily, gabapentin 600 mg every night at bedtime, and Kenalog cream twice daily for diabetic neuropathy. Her medications for hypertension and cholesterol were lisinopril 40 mg tablet once daily, Lipitor 20 mg tablet every night at bedtime, and clonidine tablet twice daily. She was taking omeprazole for her acid reflux. She occasionally took aspirin 81 mg and Tylenol 325 mg tablets for her arthritis pain. Her social history was significant for one cigarette per day. She did not drink alcohol. She had worked as a machine operator but was currently retired.

On routine follow-up examination, she was in good general health. There were no neurologic findings. VA in the right eye (OD) was 20/25 and OS 20/40. Her IOP values were 12 and 17 mmHg on latanoprostene bunod (Vyzulta) OU every night at bedtime. She was also taking Restasis OU twice a day for her dry eyes. Slit lamp examination revealed clear corneas, well-placed glaucoma tube implants OU in the anterior chambers, intraocular lenses, and enlarged optic nerve cupping of 0.9 OU, with normal vessels, maculae, and periphery. Her central corneal thickness (CCT) values were 562 microns (µm) and 557 µm. Visual field testing revealed worsening visual field defects OU (with an inferior arcuate defect OD and superior arcuate and inferior Bjerrum defects OS). Rhopressa was then added to OS every night at bedtime.

**Fig. 1:**
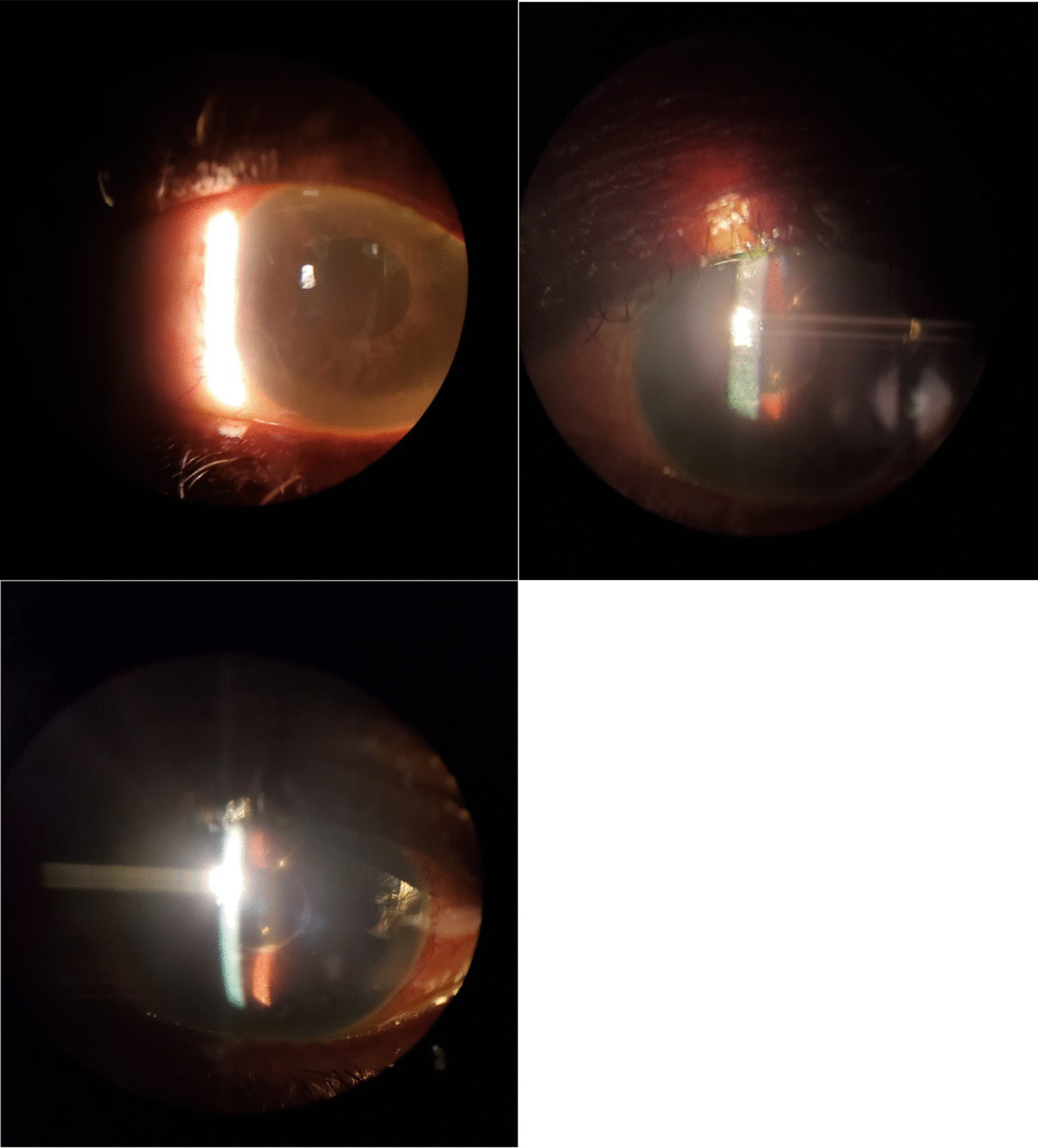
Corneal edema in the left eye 1 week after starting Rhopressa

After 5 days, the patient complained of blurry vision OS and slight pain over the past 2 days. Her VA was 20/20 OD and 20/200 OS. IOPs were 12 and 7 mmHg. Slit lamp examination revealed 2+ conjunctival injection OS and 2+ corneal edema with Descemet folds OS (Fig. 1). Her anterior chamber was deep and quiet. Optic nerve cupping was 0.9 OU. Her fundus examination was otherwise normal. CCT values were 549 µm OD and thick at 808 µm OS (Table 1). Rhopressa OS was discontinued. Sodium chloride drops and ointment OS were prescribed. Vyzulta OU every night at bedtime and Restasis OU twice a day were continued.

**Table 1:** Visual acuity, intraocular pressure, and central corneal thickness measurements during patient visits

Days	VA OD	VA OS	IOP OD (mmHg)	IOP OS (mmHg)	CCT OD (µm)	CCT OS (µm)
Baseline	20/26	20/46	12	17	562	557
Rhopressa started	20/20	20/200	12	7	549	808
Rhopressa discontinued week 2	20/20	20/40	15	17	549	589
Week 3	20/20	20/30	12	13	560	590
Month 2	20/20	20/70	7	10	571	618
Month 3	20/20-2	20/70	11	11.5	579	633
Month 4	20/20	20/30	11	14	542	560

The patient presented with a significant increase in CCT OS. *VA* visual acuity, *OD* right eye, *OS* left eye, *CCT* central corneal thickness, *IOP* intraocular pressure

The patient returned the following week with improved vision OS. She reported persistent mild blurred vision OS but had significant improvement in pain. Her VA was 20/20 OD and 20/40 OS. Her IOP values were 15 and 17 mmHg. CCT OS was significantly improved to 589 µm.

**Fig. 2:**
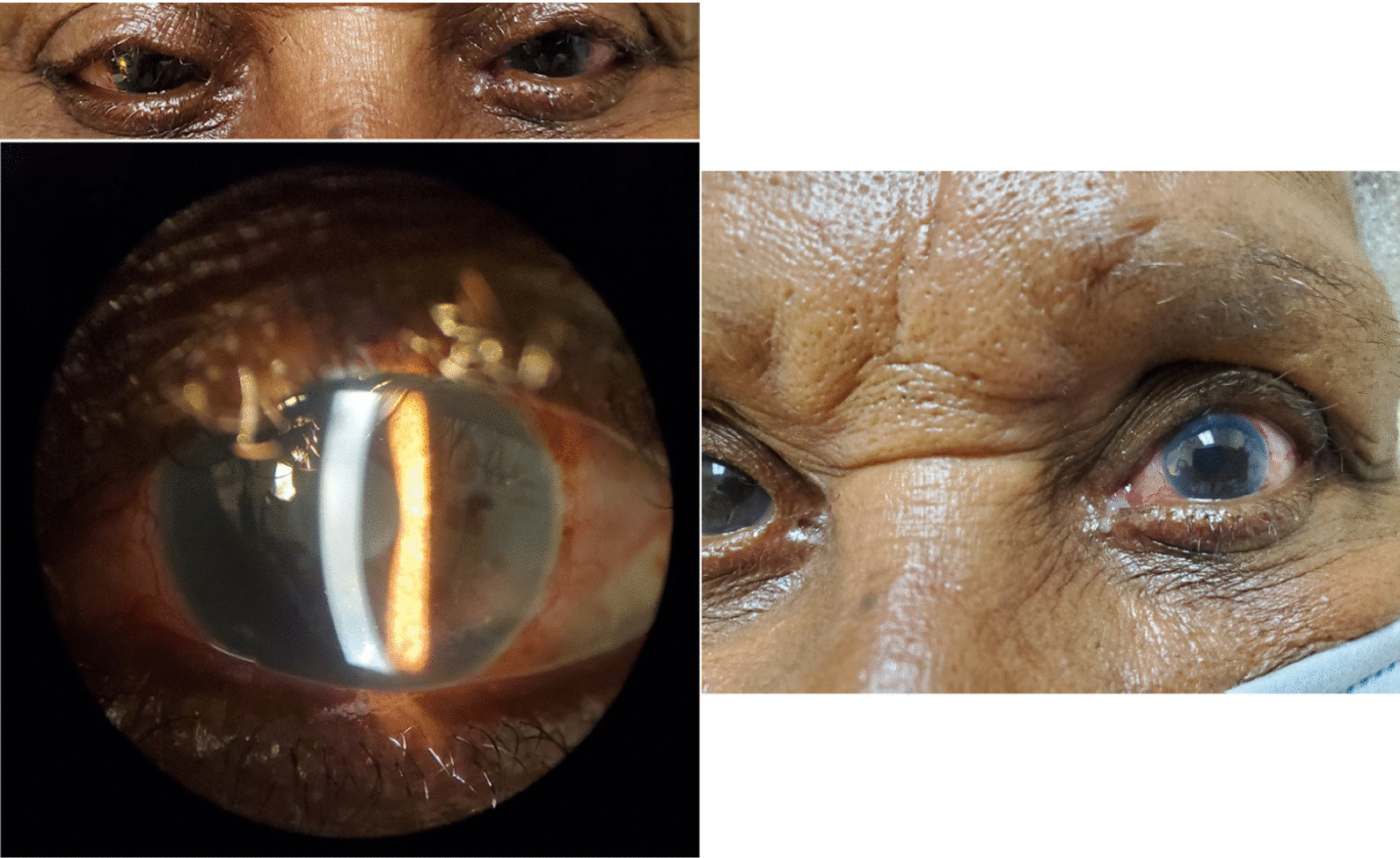
Improvement in corneal edema in the left eye upon discontinuation of Rhopressa after 2 weeks from baseline

Follow-up visits with our patient showed controlled IOP; however, she had mild increases in CCT. Over a 2-month period, the CCT values OS increased 30 µm. By month 3, her VA had worsened to 20/70, and her CCT increased to 633 µm. Topical loteprednol drops were prescribed OS every 2 hours. After 2 weeks, she improved significantly. The topical steroids were tapered. The patient had significant improvement and fortunately made a complete recovery by month 4 of discontinuation of Rhopressa (Fig. 2).

## Discussion and conclusions

Our patient presented with severe corneal edema OS 5 days after starting Rhopressa, resulting in an immediate decrease in VA and significant corneal edema. Discontinuation of Rhopressa with the addition of sodium chloride and topical steroids resulted in complete resolution of corneal edema. In contrast to other studies, our patient had no known history of corneal edema or uveitis. It is intriguing that she would react so adversely to Rhopressa. Previous research has even supported the use of ROCK inhibitors for corneal healing and reduction of corneal edema [5]. Here we see the opposite.

The patient had a drastic increase in CCT from 549 to 808 µm along with a drop in VA OS from 20/40 to 20/200. Such drastic changes in vision and CCT are unexpected. It is undeniable that Rhopressa is an effective medication for lowering IOP, as observed in our clinical findings (Table 1). Adverse events described by Aerie Pharmaceuticals have shown that conjunctival injection is a common reason for discontinuation of Rhopressa, and that decreased VA is not uncommon. However, severe corneal edema has not been reported. While there are many potential implications of ROCK inhibitors, more studies must be conducted to examine the full range of capabilities along with the effects on the cornea, specifically with Rhopressa. Our patient had no underlying corneal issues except for mild dry eye treated with Restasis; nevertheless, she had a severe reaction to Rhopressa.

The effect of ROCK inhibitors on the cornea continue to baffle researchers. Okinawa *et al*. showed significant improvement in patients with late-onset Fuchs’ dystrophy with the ROCK inhibitor Y-27632 [6]. As described earlier, some cases showed that continued Rhopressa use decreased corneal edema [8]. More evidence is needed to understand the effects of this medication on the cornea.

This case report documents the VA, IOP, and CCT values throughout the course and after discontinuation of Rhopressa treatment. While we did not observe any previous corneal abnormality, there is a possibility that her previous intraocular surgeries, including cataract extraction and glaucoma tube implants, may have predisposed her to corneal endothelial dysfunction. There is also the possibility that concomitant use of Vyzulta (Bausch & Lomb) with Rhopressa played a role in the development of her corneal edema.

We have presented a case of corneal edema acutely induced by Rhopressa, which resolved upon discontinuation of this medication and initiation of sodium chloride and topical steroids. While previous cases reported patients with a prior history of corneal edema, our patient did not have any preexisting corneal problems. Following discontinuation of Rhopressa, our patient’s CCT values returned to normal. Prior studies have demonstrated Rhopressa’s beneficial effects on the cornea. Further investigation is needed to understand the mechanism of corneal edema due to ROCK inhibitors.

## Data Availability

Data and materials are available upon request.

## Abbreviations

CCT: Central corneal thickness
DSAEK: Descemet’s stripping automated endothelial keratoplasty
FDA: Food and Drug Administration
IOP: Intraocular pressure
mmHg: Millimeters of mercury
OD: Right eye
OS: Left eye
OU: Both eyes
ROCK: Rho-associated protein kinase
SLT: Selective laser trabeculoplasty
µm: Microns
VA: Visual acuity
